# Topical prebiotic nitrate: can extending the ‘hang-time’ in the mouth improve oral-vascular health outcomes?

**DOI:** 10.1038/s41522-024-00527-3

**Published:** 2024-07-18

**Authors:** Juliana Green, Shawn J. Green

**Affiliations:** 1 Lundquist Institute, Harbor UCLA Medical, Torrance, CA USA; 2 FMC Corp, Philadelphia, USA

**Keywords:** Dental conditions, Biofilms, Microbiome, Infectious-disease diagnostics

Both oral and systemic health outcomes may improve by simply extending the residence or ‘hang time’ of prebiotic nitrate formulations in the oral cavity. This may be particularly helpful in individuals where salivary flow of nitrate is limited, as in the case of dry mouth, or those with cardiometabolic complications that exhibit dysbiosis and low nitric oxide (NO) bioavailability because of poor dietary nitrate compliance.

The thoughtful in vitro studies of Mazurel et al. and Rosier et al. shows that nitrate admixed with saliva from periodontal and healthy subjects shifts the microbiome away from disease-associated to health-associated nitric oxide-promoting microbiota. Contemporaneously, a slow-release nitrate formulated chewing gum or lozenge in healthy subjects reinforced the in vitro biofilm observations and the beneficial microbiome shift that appears to occur acutely and at low dosing with prebiotic nitrate.

Dietary nitrate (NO_3_), rich in leafy greens and the DASH Diet, is a vital micronutrient that sustains systemic NO bioavailability. NO, an ephemeral lipophilic gas, is produced by a tightly regulated family of L-arginine-dependent nitric oxide synthases found in nearly every cell type. From regulating vascular tonality and oxygen delivery to serving as an effector molecule during an immune response, NO plays a multitude of roles in human health and disease^1^. Unfortunately, Nitric Oxide Synthase (NOS) activity, especially in endothelial cells, wanes with age. Such a loss is believed to contribute to chronic disease^1–3^.

However, a redundant NO pathway exists, the entrosalivary loop, which is primary driven by NO_3_-rich dietary sources. Here, NO is restored through various redox reactions beginning with the reduction of dietary NO_3_ to nitrite (NO_2_) to NO as well as a mix of nitrogen oxide intermediates, such as long-lived S-nitrosothiols, a storage pool for NO^1–3^.

A requisite step in the bioconversion of NO_3_ to NO_2_ is catalyzed by a yet-to-be fully understood community of nitrate-reducing bacteria in the mouth^3^. The systemic importance of this NO-promoting, nitrate-reducing community is best highlighted with an increase in blood pressure after the oral cavity is exposed to mouthwash resulting in dysbiosis with a concomitant decrease in NO bioavailability and elevated blood pressure^2^.

Several clinical studies have demonstrated the anti-hypertensive effects of NO through the ingestion of 4–11 mM NO_3_-rich vegetable juice or encapsulated nitrate salt, such as potassium nitrate^4^. Upon swallowing the nitrate-containing juices or capsules, NO_3_ is absorbed in the gut and approximately 1/4 of the total NO_3_ circulates back to the mouth where NO_3_ is selectively concentrated in the salivary glands and subsequently secreted into the mouth affording both systemic and oral benefit. As to the oral benefit, a significant reduction in caries and counts of *Streptococcus mutans* and *Lactobacillus spp*. was found in patients with high salivary NO_3_^5^. Likely through the local production of NO, the reduced-NO_2_ in the mouth beginning with the bioconversion from dietary NO_3_, inhibits growth of *S. mutans*, *Lactobacillus sp., Actinomyces naeslundii*, as well as, periodontal disease-associated pathobiont *Fusobacterium sp., Eikenella corrodens* and *Porphyromonas gingivalis*^6,7^. Reportedly, the increase in salivary NO_3_ contributes to the overall protective effect against periodontal-associated pathobiont affecting both hard and soft oral tissues^8^. Sulfate-reducing bacteria, *Desulfovibrio spp*., implicated in bad breath was also found to be inhibited by NO_3_^9^.

Mazurel et al. reported that 5 mM NO_3_ decreased the biofilm quantity and dysbiosis index in an in vitro biofilm assay consisting of subgingival plaque derived from periodontal patients^10^. A small synbiotic response was also observed with the addition of the nitrate-reducing probiotic, *Rothia aeria*, in the presence of 5 mM NO_3_ resulting in further elevation of the health promoting NO precursor, NO_2_. And the addition of 50 mM NO_3_, in the absence of nitrate-reducing probiotic, further decreased periodontitis-associated species such as *Fusobacterium nucleatum*, *Tannerella forsythia*, among other pathogenic species.

Using the same in vitro biofilm assay, Rosier, Mira and colleagues demonstrated that the addition of 6.5 mM NO_3_ to saliva of healthy subjects increased the oral-health-associated NO_3_-reducing genera *Neisseria* and *Rothia* with a lowering of periodontitis and halitosis-associated *Porphyromonas*, *Fusobacterium, Leptotrichia, Avoprevotella, Tannerella and Treponema*, and the caries-associated *Oribacterium*^11^. As such, NO_3_ has emerged as a prebiotic in reversing oral dysbiosis.

**Fig. 1:**
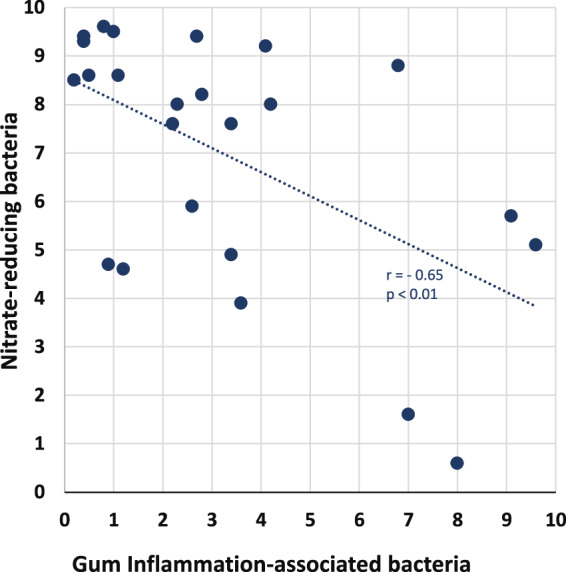
Scoring of nitrate-reducing bacteria and gum inflammation-associated bacteria communities from saliva of periodontally healthy individuals. Shotgun metagenomic sequencing was used to look at all the DNA present in the saliva microbiome samples mapped to a database of functional annotated genes. Saliva was collected and tested according to the methodology by Bristle Oral Health measuring the abundance of these bacteria using an in-house bioinformatics pipeline and then compared those abundances across thousands of samples. The scoring method is based on a priori knowledge of selected bacteria in context of functional annotated genes and takes into consideration the relative abundance of each bacterial strain to compute a nitrate-reducing and risk score for gum disease-associated bacteria where 0 is the lowest and 10 is the highest abundance. The nitrate-reducing community consisted of *Rothia mucilaginosa, Neisseria flavescens, Veillonella dispar, Veillonella atypica, Haemophilus parainfluenzae* and the gum inflammation-associated cluster included *Tannerella forsythia, Treponema socranskii, Fusobacterium periodonticum, Porphyromonas gingivalis, Streptococcus constellatus, Fusobacterium peridoncum, Fusobacterium nucleatum, Parvimonas micra*.

Saliva from a mixed population of healthy subjects were assessed for the relative abundance of nitrate reducers in comparison to an inflammation-associated bacteria cluster. As shown in Fig. 1, the nitrate-reducing community correlated negatively with inflammation-associated cluster (*r* = −0.65, *N* = 23, *p* < 0.01; see list of communities for nitrate-reducers and disease-associated bacteria in figure legend). A subset of 9 healthy individuals from this cohort were subsequently identified as low nitrate-reducers as determined by the absence or <20 μM salivary NO_2_ and consented to chewing a prebiotic NO_3_-ascorbic acid formulated chewing gum.

**Fig. 2:**
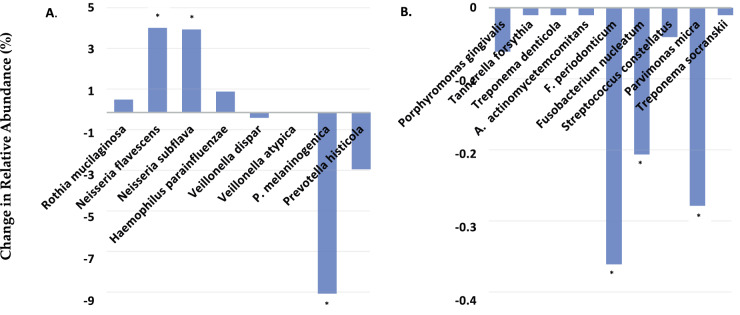
Change in the relative abundance of pre and post topical administration of a prebiotic nitrate formulated chewing gum in healthy subject with low pre-gum salivary nitrite, as determined by the absence or <20 μM salivary nitrite (Saliva Nitric Oxide Test Strips by MyFitStrips). **A** The change of the saliva microbiome of pre- and post-chewing of selected bacteria species with >1% relative abundance of the pre-chewing gum sampling and with a prior knowledge of nitrate-reducing capacity. **B** The change or difference in abundance of pre- and post-chewing in healthy subjects pre-selected with a prior knowledge of disease-associated attributes, albeit this community was well less than 1% abundance in pre-saliva sampling. Whole genome shotgun metagenomic sequencing was used to looks at all DNA present in the saliva microbiome samples from pre- and post-chewing. Saliva was collected and tested according to the methodology by Bristle Oral Health. *p < 0.05 between pre- and post-chewing after 4–5 h from the initial piece of gum of a total of 3 dispersed over this time.

Figure 2A, shows the difference in the saliva microbiome of pre- and post-chewing of the selected bacteria species with >1% relative abundance of the pre-chewing gum sampling and with a prior knowledge of nitrate-reducing capacity. Pre- and post-chewing saliva samples were collected for metagenomic sequencing of all the DNA present in the saliva samples and mapped to a database of functional annotated genes. Individuals chewed a 0.62 mM prebiotic NO_3_ formulated gum for 5–10 min per session. This was repeated throughout day for a total of 3 chewing sessions. Each chewing session was separated by ~2.5 h for a total of 1.86 mM NO_3_ for the day. Post-saliva samples for metagenomic sequencing were collected 5–6 h after exposure to the first gum.

After intermittent exposure to NO_3_ gum, a significant increase was observed in the oral health-associated nitrate-reducing *Neisseria flavescens, Neisseria subflava* (both *p* < 0.05), *Haemophilus parainfluenzaea*, and *Rothia mucilaginosa*. A significant decrease in *Prevotella melaninogenica* and *Prevotella histicola* (both *p* < 0.05) was also observed with an insignificant decrease in both *Veillonella sp*. within 1 day. Periodontitis-disease-associated species were also searched for, albeit this community was well less than 1% abundance in pre-saliva sampling. Nonetheless, Fig. 2B shows that periodontitis- associated bacterial, to include *Porphyromonas gingivalis, Fusobacterium nucleatum*, and *Tannerella Forsythia* were sensitive to the prebiotic NO_3_ chewing gum resulting in a reduction during this short period.

These initial observations suggest that intermittent chewing of a dietary NO_3_ source over the course of hours begins to shift the oral microbiome to a nitric oxide favorable microbiota with a concomitant decrease in disease-associated bacteria with as little as 1.86 mM NO_3_ within 1 day. These observations are the basis of a larger clinical study examining the effects of intermittent chewing of a low-dose prebiotic NO_3_-ascorbic acid formulation in a slow-release chewing gum for 21 days on gingival inflammation and systemic endothelial function in periodontal patients that has received ethical approval at the University Maryland Dental School, Baltimore, MD.

Previous studies have shown that a daily bolus ingestion of drinking a beetroot or lettuce juice concentrate consisting of 3–12 mM NO_3_/day over the course of 10 days to 6 weeks enriched a NO-favorable microbiome and improved NO-mediated oral-systemic health^4^. Vanhatalo et al. showed that 10 days of drinking 6.2 mM NO_3_/day increased the abundance of *Rothia spp* (+127%) and *Neisseria spp* (+351%) with a corresponding decrease in *Veillonella spp* (−60%) and *Prevotella melaninogenica* (−60%) with an increase in NO_2_ and reduced systemic blood pressure^12^. After 6 weeks of drinking 6.7 mM NO_3_ beet juice daily, Velmurugan et al. observed improvement in endothelial function with a corresponding increase in *Rothia mucilaginosa* and *Neisseria flavescens*^13^. And Jockel-Schneider et al. observed in periodontal recall patients a marked decrease of gingival inflammation with a corresponding increase in *Rothia spp* and *Neisseria spp* after consuming 3 mM of NO_3_-rich lettuce juice daily for 2 weeks^14^.

By simply extending the residence or ‘hang time’ of prebiotic NO_3_ formulations in the oral cavity may acutely improve both oral and cardiovascular health outcomes. As such, hypertensives with poor dietary NO_3_ compliance or where salivary flow of NO_3_ is limited, as in the case of Xerostomia patient, may benefit the most. Restoring a NO-favorable oral microbiome may emerge to be equally beneficial to smokers, ex-smokers and those with cardiometabolic complications who all exhibit dysbiosis and low NO bioavailability. The latter is reinforced by Goh et al. showing a causal relationship between elevated *Neisseria flavescens* and *Haemophilus parainfluenzae* with lower insulin resistance and blood pressure^15^. Extended chewing-time of a prebiotic nitrate-ascorbic acid-polyphenol formularies delivered through a functional chewing gum, lozenge, or slowly munching a whole food plant-based source, such as a rocket-beet-citrus salad, may emerge to be more effective and durable in NO-mediated oral-systemic health outcomes than simply drinking NO_3_-rich beet juices or swallowing a NO_3_ pill. At a minimum, by enhancing a nitric oxide favorable oral microbiome with prebiotic nitrate formulated chewing gums or slow-release lozenge will potentiate heart healthy foods and diets that improve nitric oxide bioavailability^16^.
